# Synergistic Effect of Light and Temperature on Growth and Biochemical Composition of *Chlorella sorokiniana* Cultures

**DOI:** 10.3390/foods15061087

**Published:** 2026-03-20

**Authors:** Ana Margarita Silva Benavides, Natalia Jiménez-Conejo, Giuseppe Torzillo

**Affiliations:** 1 Escuela de Biología, Universidad de Costa Rica, San Pedro de Montes de Oca, San José 11501-2060, Costa Rica; ana.silva@ucr.ac.cr (A.M.S.B.); natalia.jimenezconejo@ucr.ac.cr (N.J.-C.); 2 CIMAR—Centro de Investigación en Ciencias del Mar y Limnología, Universidad de Costa Rica, San Pedro de Montes de Oca, San José 11501-2060, Costa Rica; 3 CNR-Istituto per la Bioeconomia, Via Madonna del Piano 10, Sesto Fiorentino, I-50019 Florence, Italy

**Keywords:** *Chlorella sorokiniana*, growth productivity, light intensity, temperature, fluorescence, biochemical composition, amino acids, fatty acids

## Abstract

This study investigated the combined effects of light intensity and temperature on the growth and biochemical composition of *Chlorella sorokiniana* (Chlorophyceae) under controlled laboratory conditions. The cultures were exposed to two continuous photon flux densities (100 and 200 µmol m^−2^ s^−1^) and six different temperatures (20, 25, 30, 35, 40, 45 °C). At a light intensity of 100 µmol m^−2^ s^−1^, the highest attained volumetric productivity was 16.92 mg DW L^−1^ h^−1^ at 25 °C, resulting in a mean final biomass density of 3.25 g DW L^−1^ after 8 days of cultivation. In contrast, at 200 µmol m^−2^ s^−1^, there were notable differences in growth performance, with maximum biomass volumetric productivity reaching 31.4 mg DW L^−1^ h^−1^ at 30 °C and a final biomass density of 6.08 g DW L^−1^. The optimal temperature for growth depended strongly on light intensity. Cultures at 20 °C thrived at 100 µmol m^−2^ s^−1^ but showed negligible growth at 200 µmol m^−2^ s^−1^. No growth occurred at 45 °C under either light intensity. Furthermore, temperature significantly affected biomass composition, affecting both fatty acid and amino acid profiles. These findings provide valuable insights for optimizing the cultivation of *C. sorokiniana* outdoors.

## 1. Introduction

Microalgae are important primary producers in both freshwater and marine ecosystems. They use light energy and carbon dioxide to produce biomass, playing a key role in global carbon cycling. Among microalgae, *Chlorella sorokiniana* is a unicellular, spherical, non-flagellated green microalga with a diameter of 2–10 μm, belonging to the phylum Chlorophyta [1].

*Chlorella sorokiniana* was first isolated in 1953 by Sorokin and was initially described as a thermotolerant mutant strain of *Chlorella pyrenoidosa* [2,3]. However, during the late 1980s and early 1990s, advances in molecular taxonomy, particularly chloroplast 16S rDNA and nuclear 18S rRNA profiling, led to its reclassification as a distinct species [4,5].

The biomass of *C. sorokiniana* has been widely proposed as a source of lipids for biodiesel production [6], as well as for food supplements [7] and for metabolites with pharmacological antioxidant activity [8]. Its high protein content and amino acid composition also make it a promising ingredient for human food [9]. In addition, it is a valuable source of high-value compounds, including fatty acids and pigments (e.g., chlorophyll) [10,11,12], and a sustainable substitute in aquafeeds [13]. Moreover, *C. sorokiniana* biomass has been evaluated as a dietary supplement that improves growth performance, antioxidant status, and immune response in rainbow trout, and it is also used as feed for rotifers, which serve as live food for marine fish larvae [14]. Finally, *C. sorokiniana* has been extensively investigated for wastewater treatment applications, often coupled with biodiesel production, highlighting its potential within circular bioeconomy frameworks [15,16].

Light and temperature are two fundamental environmental factors governing the autotrophic growth of microalgae, as they strongly influence biomass productivity [17]. Although the effects of individual factors—particularly temperature—on *Chlorella sorokiniana* have been widely studied, information regarding the interactions between light and temperature remains limited [18]. These two environmental factors are especially critical for the cultivation of *C. sorokiniana* outdoors, where light intensity can fluctuate by an order of magnitude over the course of a day. In outdoor reactors, particularly in desert environments, a frequent scenario involves low temperatures combined with rapidly increasing light intensities during the early morning hours. Under such conditions, light intensity often rises faster than reactor temperature, rendering the photosynthetic apparatus susceptible to photoinhibition and consequently reducing daily productivity [19]. Light spectrum is another important factor that can affect both the growth and the biomass composition of microalgae [20].

The ability of *Chlorella sorokiniana* to acclimate to fluctuating environmental conditions makes it a valuable model organism for studying the interplay between environmental drivers and biomass composition [21]. Despite this adaptive capacity, the average productivity of most industrially relevant microalgal strains, including *Chlorella*, remains significantly lower than their theoretical maximum. This discrepancy underscores the importance of identifying key factors limiting biomass yield and of removing physiological bottlenecks as part of domestication strategies to enhance the industrial viability of algal-derived bioproducts.

To gain further insight into the productivity and biomass composition of *Chlorella sorokiniana*, cells were exposed to combinations of temperatures (20–45 °C) and white-light intensities (100 and 200 µmol m^−2^ s^−1^). Culture performance was evaluated using growth measurements, biochemical analyses of biomass composition, and chlorophyll fluorescence quenching to assess the physiological status of cultures under different light and temperature regimes.

## 2. Materials and Methods

### 2.1. Organism and Culture Conditions

*Chlorella sorokiniana* was obtained from the UTEX Culture Collection of Algae (UTEX 1230), University of Texas at Austin. The inoculum was grown in K3 medium [22] in glass columns (4.8 cm internal diameter, 5 cm external diameter) with a 400 mL working volume, at 30 °C, and bubbled with a mixture of air/CO_2_ (97:3, *v*/*v*). Chemicals were purchased from Sigma Aldrich. The pH of the culture medium was maintained at 7.0 ± 0.2. Cultures were illuminated at 70 μmol m^−2^ s^−1^ on the surface of the culture vessel using cool-white, fluorescent light (Dulux L, 55W/840, Osram, Italy).

### 2.2. Experimental Conditions

The *C. sorokiniana* were grown in batch cultures for 8 days. All experiments were conducted in triplicate using 400 mL glass columns with an internal diameter of 4.8 cm. Experiments were carried out under continuous illumination at 100 and 200 μmol m^−2^ s^−1^ and at 6 temperatures (20, 25, 30, 35, 40, 45 °C). Therefore, a completely randomized 2 × 6 factorial design was used, with 2 light intensities (100 and 200 PFD) and temperature (20–45 °C) as the experimental factors. Each treatment combination was performed in triplicate. Individual cultures constituted experimental units. Irradiance was measured at the midpoint of each empty glass column using a cosine-corrected flat quantum sensor (Licor quantum), connected to a light meter (LI-250 A). Each tube was bubbled with a sterilized air/CO_2_ (97:3, *v*/*v*) mixture at 0.2–1.0 vvm (volume of gas per volume of liquid per minute), and the flow was increased according to the rise in the cell density to guarantee sufficient mixing (uniform light exposure) and CO_2_ supply. The pH of the cultures was frequently monitored to maintain it near the optimal value (7.0). The glass columns were inoculated with an initial biomass dry weight of about 100 mg L^−1^. The medium and glass columns were sterilized in an autoclave at 120 °C for 45 min to prevent contamination during the growth experiments. After 48 and 96 h of cultivation, the concentrations of the main nutrients, N and P, were restored to their initial values by adding sterile stock solutions of the corresponding salts. Mg and S concentrations were not monitored, as their amounts in the culture medium were sufficient to achieve a biomass dry weight greater than 7 g L^−1^. Indeed, Mg and S account for less than 0.5% of the biomass dry weight. Before sampling the culture, evaporation was compensated for by adding distilled sterile water.

### 2.3. Dry Weight Determination

Biomass was measured in triplicate using 10 mL or 5 mL samples, depending on the culture density. Samples were collected from columns at 24 h intervals using sterile pipettes to determine the increase in dry cell weight. Each sample was filtered through pre-weighted 0.45 μm pore-size membranes and then dried at 105 °C for 3 h. The filters were maintained in a desiccator to equilibrate at room temperature, then weighed. Mean biomass productivity (mgDW L^−1^ h^−1^) was calculated according to the equation (*x*_1_ − *x*_0_)/∆*t*, where *x*_1_ and *x*_0_ (mgDW L^−1^) were the dry weight at intervals of 24 h (∆*t*).

### 2.4. Chlorophyll Fluorescence Measurements

Daily measurements of the fluorescence parameter F_v_/F_m_, the maximum photochemical yield of PSII, and ETR (electron transfer rate) were performed with a portable pulse-amplitude-modulation fluorometer (PAM 2100, H. Walz, Effeltrich, Germany). For this purpose, 0.5 mL of the algal samples was taken from the culture tubes and incubated in the dark for 15 min. Minimum PSII fluorescence (F_0_) was measured with a low-intensity modulated measuring beam (0.3 μmol photons m^−2^ s^−1^) from light-emitting diodes (peak wavelength at 650 nm, frequency 600 Hz). A saturating light pulse was used to reach the F_m_ level in the dark-adapted cells. To perform ETR vs. PFD (photon flus density) curve, a series of stepwise increasing irradiance intensities (LEDs, 0–636 μmol m^−2^ s^−1^) provided by a PAM-2100 were automatically applied at 20 s intervals to obtain the light-adapted fluorescence level F’ (steady-state fluorescence yield in the light), and at the end of each step a saturating pulse (>6000 μmol photons m^−2^ s^−1^, 0.6 s duration) was triggered to reach the maximum fluorescence level F_m_′ (steady-state maximum fluorescence in the light). The effective PSII photochemical quantum yield in the light, YII, was determined as (F_m_′ − F′)/F_m_′ in the light-adapted state at the respective irradiance level. The electron transport rate at each light step was calculated by the PAM-2100 software.

We used the non-linear least-squares regression model [23] to fit the ETR/PFD data to estimate the maximum electron transport rate (ETR_max_), α_ETR_ (i.e., the initial slope of the curve), which is proportional to the quantum yield of photosynthesis. The I_k_ (i.e., the saturation irradiance) was given as an intercept between α_ETR_ and ETR_max_.

### 2.5. Chemical Composition and Fatty Acid Methyl Esters (FAMEs) Analysis of Biomass

Biomass was harvested by using a refrigerated Beckman Coulter centrifuge (model Avanti). Samples were lyophilized and stored at −80 °C prior to chemical analysis of carbohydrates, proteins, and lipids. The carbohydrate content of the biomass was measured using the phenol-sulphuric acid method [24], with D-glucose as a standard.

### 2.6. Chemical Analysis of Protein in the Biomass

Protein content was analyzed on fresh biomass by the Folin-phenol method [25], after hydrolysis with 1N NaOH for 10 min in an ultrasonicator, followed by heating to 100 °C for 5 min. Lipids were extracted from the lyophilized pellet as described in [26]. Fatty acid methyl esters were prepared by direct esterification of lipid extracts. The component fatty acids were separated and identified using GC (HRGC 5300 Mega series, Carlo Erba, fitted with a fused-silica capillary column, 60 m/0.25 mm internal diameter, TR-FAME, 0.25 µm film thickness, and a flame ionization detector (FID).

### 2.7. Protein Amino Acid Compositions

Amino acid composition was determined according to the following procedure [27]. The microalgal lyophilized biomass was subjected to hydrolysis in a vapor phase in a nitrogen atmosphere at 114 °C for 24 h with 200 μL 6 mol L^−1^ HCl solution with 0.1% phenol to prevent halogenation of Tyr. The samples were then re-suspended in 50 μL 0.1 mol L^−1^ HCl and subjected to the derivatization process. Internal standard (25 μL Nval 5 mmol L^−1^) and NaOH (393 μL 0.5 mol L^−1^) were introduced into AccQ _*_ Tag Ultra borate buffer. For each sample, 10 μL of solution was collected and placed in a glass vial insert with 70 μL of a solution of 0.0417 mmol L^−1^ Nval/0.0655 mmol L^−1^ NaOH in AccQ _*_ Tag borate buffer and 20 μL AccQ _*_ Tag Ultra reagent. For each cycle of analysis, a calibration standard (CS) containing 17 amino acids and Nval at a concentration of 10 pmol μL^−1^, except Cys, present at a concentration of 5 pmol μL^−1^, was used. This solution was prepared with 40 μL 2.5 mmol L^−1^ amino acid standard solution and 20 μL 5 mmol L^−1^ Nval in 940 μL Milli-Q water. CS (10 μL) was placed in the glass insert with 70 μL borate buffer and 20 μL AccQ _*_ Tag Ultra reagent. Chromatography was performed with a Waters (USA) Acquity UPLC instrument equipped with a BEH C18 column (2.1 × 100 mm, 1.7 μm particle size) and a UV detector (detection wavelength 260 nm). Two different eluents were used: A (AccQ _*_ Tag Ultra eluent A, diluted in the ratio 1:20 with Milli-Q water) and B (AccQ _*_ Tag Ultra eluent B). The gradient elution program was: 0.54 min, 0.1% B; 5.74 min, 9.1% B; 7.74 min, 21.2% B; 8.04 min, 59.6% B; 8.05 min, 90% B; 8.73 min, 0.1% B; 9.50 min, 0.1% B. The column temperature was 55 °C, the flow rate was 0.7 mL min^−1^, and the injection volume was 1 μL (partial loop). All samples were mixed, heated to 55 °C for 10 min, and injected for Ultra-Performance Liquid Chromatography (UPLC) analysis. Compared with conventional HPLC instruments, UPLC technology offers greater sensitivity and resolution, reduced analysis time and solvent consumption, owing to the smaller packing-particle diameter (1.7 μm) and the higher pressure in the columns. UPLC is the preferred methodology for amino acid determination.

### 2.8. Statistical Analysis

Results were analyzed using a factorial ANOVA to assess the effects of light and temperature on biomass productivity and biochemical components (proteins, carbohydrates, and lipids). Tukey’s test was used to assess the statistical significance of the effect of light and temperature, and their interaction on productivity, carbohydrates, proteins, and lipids.

## 3. Results

### 3.1. Productivity of Cultures

**Figure 1:**
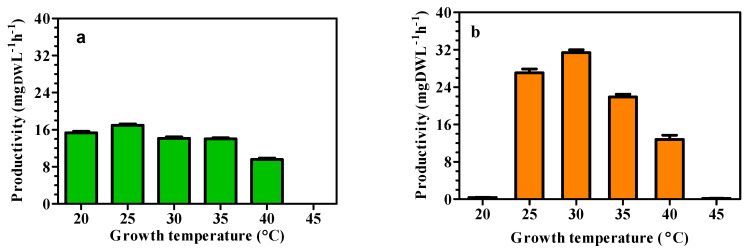
Mean productivity (mgDWL^−1^ h^−1^) of *C. sorokiniana* UTEX 1230 cultures grown under different temperature and light intensity combinations for 8 days. Data are the average productivities recorded over 8 days of batch cultivation carried out in triplicate. (**a**) PFD = 100 and (**b**) PFD = 200 μmol m^−2^ s^−1^ (*n* = 3).

Figure 1 shows the mean productivities achieved in cultures of *C. sorokiniana* grown at different temperatures and irradiated with 100 μmol m^−2^ s^−1^ (Figure 1a) and 200 μmol m^−2^ s^−1^ (Figure 1b).

Optimal culture productivity was observed at 25 °C under 100 μmol m^−2^ s^−1^ (Figure 1a) and at 30 °C under double irradiance (Figure 1b). Therefore, we observed a 5 °C shift in the optimal growth temperature at the two tested irradiances. Moreover, at 100 μmol m^−2^ s^−1^, the difference in productivity between the different temperatures was lower (Figure 1a) than that at 200 μmol m^−2^ s^−1^ (Figure 1b). At 20 °C and 200 μmol m^−2^ s^−1^, productivity was negligible (Figure 1b) because of the combination of high irradiance and suboptimal temperature, which inhibited growth. Both at 100 and 200 μmol m^−2^ s^−1^, no growth was detected at 45 °C, indicating that 40 °C is the upper limit for appreciable productivity of *C. sorokiniana*. The ANOVA (factorial) analysis of data biomass productivity showed statistically significant effects of light (F = 208.00; *p* < 0.001), temperature (F = 706.60; *p* < 0.001), and the interaction between the two factors (F = 270.10; *p* < 0.001), indicating that the effect of light depended on temperature.

### 3.2. Chlorophyll Fluorescence

**Figure 2:**
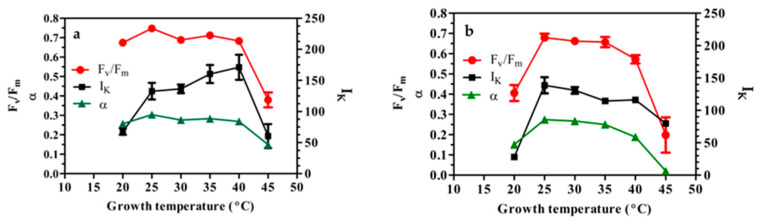
Changes in the maximum photochemical yield of PSII (F_v_/F_m_), initial slope α, and saturation irradiance I_k,_ measured in *C. sorokiniana* cells growing at different temperatures, at irradiance of 100 (**a**) and 200 (**b**) µmol m^−2^ s^−1^. Data are the mean productivity values recorded over 8 days of batch growth. The experiments were carried out in triplicate (*n* = 3).

Figure 2 shows changes in the F_v_/F_m_ ratio and the main photosynthesis parameters recorded at irradiances of 100 (Figure 2a) and 200 μmol m^−2^ s^−1^ (Figure 2b) and at different growth temperatures.

The F_v_/F_m_ ratio of cultures grown at 100 μmol m^−2^ s^−1^ remained close to the optimum value of 0.7 for microalgae culture suspensions, within a wide temperature range (from 20 °C to 40 °C); the highest value 0.744 (*p* < 0.05) was attained by the cultures grown at 25 °C; it sharply decreased to 0.356 (mean value) when cells were grown at 45 °C (Figure 2a). Under 200 μmol m^−2^ s^−1^, the F_v_/F_m_ values were significantly lower than the optimal value, both at 20 °C (mean value 0.405) and particularly at 45 °C (mean 0.11). Between 25 °C and 40 °C, the F_v_/F_m_ ratio showed a clear tendency to reduction as the temperature increased. It appeared clear that a suboptimal temperature of 20 °C coupled to higher light caused a negative synergistic effect on the maximum photochemical quantum yield of PSII (Figure 2b). The saturation irradiance I_k_ and the initial slope α showed different behaviors at 100 and 200 light irradiances. At 100 μmol m^−2^ s^−1^, both I_k_ and α showed the minimum values, with a more marked effect at 20 °C and 45 °C (Figure 2a,b). At under 100 μmol m^−2^ s^−1^ and between 20 °C and 40 °C, I_k_ and α showed a divergent behavior (Figure 2a). On the contrary, at 200 μmol m^−2^ s^−1^, both I_k_ and α followed approximately the same trend, with a sizeable increase at 25 °C; thereafter, they gradually decreased as the temperature increased to 45 °C (Figure 2b).

**Figure 3:**
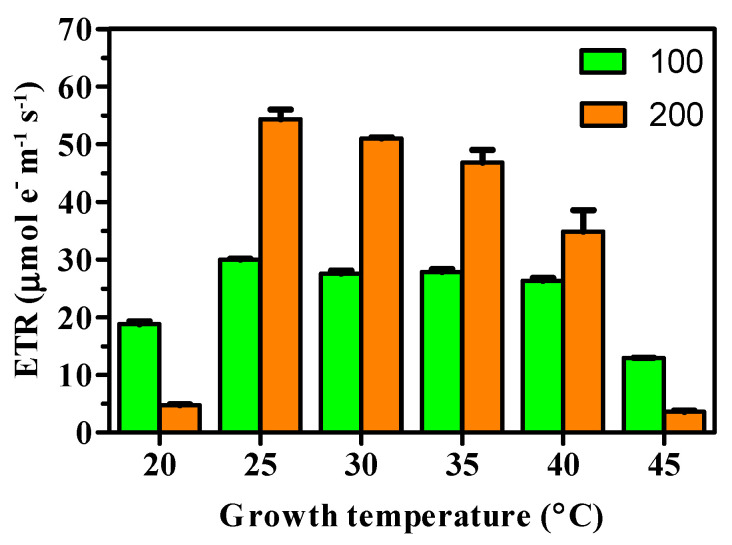
Electron transfer rate (ETR) recorded in cells of *C. sorokiniana* grown under two light irradiances and different temperatures. Data are the average productivities recorded over 8 days of batch cultivation carried out in triplicate (*n* = 3).

In Figure 3, the electron transfer rates (ETR) are shown for the different culture conditions. As observed previously for the main photosynthesis parameters, electron transfer rates showed a clear dependence on the combination of light and temperature. Under 100 μmol m^−2^ s^−1^, the ETR of cells resulted quite similar within 25 °C and 40 °C (Figure 3). At the same time, a much higher interaction effect between light and temperature was observed in cultures grown under 200 μmol m^−2^ s^−1^, particularly when they were grown at 20 °C (Figure 3).

### 3.3. Biochemical Composition of Biomass

The biochemical composition of *C. sorokiniana* biomass grown under two light irradiances and different temperatures is summarized in Table 1. A higher and stable protein content, was recorded under 100 μmol m^−2^ s^−1^ at growth temperatures ranging between 25 °C and 35 °C, while in cultures grown under 200 μmol m^−2^ s^−1^, a much larger variation in the protein content was observed, ranging from 30.3% (average) within 25 °C and 30 °C to a maximum of 57.3% at 40 °C (Table 1). No significant differences in the protein content were found at 40 °C between cultures grown at the two light irradiances. At the lowest temperature tested (20 °C), growth was attained only under 100 μmol m^−2^ s^−1^, and the protein content was the lowest value recorded among all the growth tested conditions, while at 45 °C, because of the lack of growth, no data on protein content could be gathered under either of the irradiances. The highest biomass protein content was found in cultures grown at 40 °C under both 100 μmol m^−2^ s^−1^ and 200 μmol m^−2^ s^−1^, at 58.7% and 57.3%, respectively.

Carbohydrates showed an opposite trend compared with protein. The highest carbohydrate content in the biomass was observed at 200 μmol m^−2^ s^−1^ and 25 °C (mean 41.8% of DW), peaking at 48% at 25 °C (Table 1). In cultures grown at 100 μmol m^−2^ s^−1^, carbohydrates were the predominant biochemical components only at 20 °C. At 40 °C, they were at the minimum, 16.07% and 18.00%, under 100 and 200 μmol m^−2^ s^−1^, respectively. At 45 °C, there was insufficient biomass for analysis, as the cultures did not grow.

As for lipids, there was a much narrower range of variation across the different temperatures than for protein and carbohydrate (Table 1). However, at 100 μmol m^−2^ s^−1^, lipid content was significantly higher than that observed in cultures exposed to 200 μmol m^−2^ s^−1^. At 100 μmol m^−2^ s^−1^, the lowest lipid content was observed at 40 °C, whereas at 200 μmol m^−2^ s^−1^, the lowest value was observed at 25 °C. Because there was no growth at either 20 °C or 45 °C, no analysis could be performed.

The fatty acid profile of the biomass grown at 100 μmol m^−2^ s^−1^ for temperatures of 25, 35, and 40 °C is presented in Table 2. The dominant fatty acids of *C. sorokiniana* were C16:0, C18:1 n9, C18:2 n6, and C18:3 n3. Concerning the saturated fatty acid C16:0, it was interesting to note its increase with increasing culture temperature, reaching 29.51% of total fatty acids in cells grown at 35 °C. Among unsaturated fatty acids, the C18:1 n9 (oleic acid) content did not significantly vary over the temperature range 25–40 °C (*p* > 0.05). The concentration of linoleic acid (C18:2 n6) varied from 54.40% at 25 °C to 58.20% at 40 °C (*p* > 0.05). The fatty acid C18:3n3 (α-linoleic) content was higher at 25 °C, 14.63%, and decreased significantly (*p* < 0.001) with the increase in temperature. The ratio of saturated fatty acids (SFAs) to unsaturated fatty acids (UFAs) (S/U ratio) increased with the increase in temperature, particularly when the temperature for growth was increased to 35 °C (Table 2).

Table 3 shows the amino acid profile of *C. sorokiniana* biomass grown at 100 µmol m^−2^ s^−1^ within the temperature range of 20 °C to 40 °C. Except for tryptophan, all the other essential amino acids were present. Amino acid composition did not vary much among the growth temperatures, except for lysine, tyrosine, and methionine in cells grown at 40 °C. Indeed, at this growth temperature, lysine increased by 14% relative to the average of 8.26%, tyrosine increased by 25% (mean 0.91%), and methionine increased by 33% (mean 0.51%). The increases in lysine, tyrosine, and methionine were counterbalanced by a reduction in the relative amounts of proline, leucine, and phenylalanine (Table 3).

## 4. Discussion

Microalgal cultures outdoors are rarely subjected to single stressors; instead, they experience the combined, often interacting effects of multiple environmental factors, with light and temperature among the most important. Therefore, to enable industrial algal biotechnology to benefit from laboratory findings, it is essential to examine the interplay among environmental factors that affect growth and macromolecular biomass composition. The temperature tolerance range of microalgae is usually higher below the optimal temperature but lower above it. [28]. However, this general pattern can be influenced by the light intensity. Indeed, in *Chlorella sorokiniana* grown at 200 µmol m^−2^ s^−1^, we observed a narrow range of growth below the optimal temperature (20–30 °C) and a broader range above it (30–40 °C). Whereas photochemical reactions proceed at rates that are almost independent of temperature, the rates of enzyme-mediated dark reactions decrease at lower temperatures [29]. Thus, exposure of microalgae to suboptimal temperatures may result in the absorption of more light energy than the photosynthetic apparatus can use for carbon fixation, necessitating the activation of dissipating energy mechanisms to maintain the energy balance. Low-temperature conditions increase the reduction in the PSII complex and the redox state of the quinone acceptors and may act as a signal to trigger acclimation processes [30]. Possible mechanisms may include: (a) alterations in light harvesting and primary photochemistry to reduce light energy input; (b) increased rates of cyclic or pseudo cyclic electron transport to spend energy (Mehler reaction); (c) higher rates of photorespiration, or (d) increased activity of the Calvin–Benson cycle. However, when these dissipation pathways become saturated, photoinhibition can occur, reducing photosynthetic activity and growth.

On summer mornings, outdoor light intensity often increases much more rapidly than culture temperature. This temporal desynchronization can induce light stress under suboptimal temperature conditions, thereby increasing the photosynthetic apparatus’s susceptibility to photoinhibition [19]. Supra-optimal temperature also alters the redox state of PSII acceptors and reduces the rate of photosynthetic electron transport through both photosystems [31]. Consequently, the development and selection of microalgal phenotypes capable of maintaining high growth rates under fluctuating environmental conditions are key prerequisites for strain optimization. Under low light (100 µmol m^−2^ s^−1^), productivity varied only modestly across temperatures, indicating that light limitation was the primary factor controlling growth. In contrast, under higher light (200 µmol m^−2^ s^−1^), productivity was much more sensitive to temperature, and no growth occurred at 20 °C. Measurements of PSII photochemical efficiency (F_v_/F_m_) and electron transport rate (ETR) confirmed that cells exposed to higher light and low temperature exhibited significant photo stress, consistent with previous reports [32]. A key finding of this study was a 5 °C shift in optimal growth temperature—from 25 °C to 30 °C—when light intensity increased from 100 to 200 µmol m^−2^ s^−1^, highlighting a clear synergistic effect between light and temperature. This result further emphasizes that strain evaluation and selection should be based on multifactorial conditions rather than single-variable assessments.

Mixing of the culture is an important factor that can modify both the culture temperature and the cells’ light uptake. Although the decrease in irradiance is continuous through culture depth, several zones can be described in a dense microalgae culture exposed to direct solar irradiance: (i) The surface layers in which irradiance is excessive and down-regulation of photosynthesis appears; (ii) the light saturated zone where culture irradiance is saturating and the high photosynthetic rate (P_max_) is reached; (iii) the light limited zone with maximum light use efficiency; (iv) the dark zone where the irradiance is below the photosynthesis compensation point and only respiration takes place. Microalgal cells in culture undergo a transition from high to low irradiance as culture density increases, leading to increased cell pigmentation and reduced light penetration, in turn reducing productivity. Enhancing turbulence aims to prevent cell acclimation to low-light conditions [33]. However, cell light uptake is not only modulated by incident light intensity but also by cell density, culture depth, mixing frequency, and the frequency of light–dark (L/D) cycling [34,35,36]. Thus, in ‘short’ light-path (<0.5 cm) cultivation systems, it is possible to induce fast L/D cycling of cells. A high frequency of L/D cycles, close to the turnover rate of the plastoquinone pool, promotes increased biomass productivity. While 60 Hz flash frequencies are beyond engineering feasibility, flash frequencies near 1 Hz appear attainable in thin-layer cultures [37].

The culture system can also strongly influence culture temperature. In closed photobioreactors, the morning rise in culture temperature is much faster than in open ponds. It can readily exceed the physiological optimum, necessitating a cooling system, thereby increasing biomass costs [38]. By contrast, outdoor cultures in open ponds are naturally cooled by evaporation, which is more effective as the culture velocity increases. Moreover, the culture system design can influence cell light uptake. Closed photobioreactors are usually characterized by higher surface-to-volume ratios (A/V) than open pond systems. Therefore, reactors with a higher A/V ratio will receive a higher light energy per unit volume of culture [39]. As a result, selecting an appropriate culture system is another critical issue for the successful exploitation of a given microalgal strain.

*Chlorella sorokiniana* exhibited substantial variation in macromolecular biomass composition in response to different combinations of light intensity and temperature. This pronounced metabolic plasticity represents an important advantage, enabling targeted manipulation of growth conditions to promote the synthesis of specific biochemical compounds. Protein content was highest in cultures grown under low light and moderate temperatures (25–35 °C), whereas carbohydrate accumulation was favored under higher light intensities within the same temperature range. Biomass rich in protein is particularly suitable for health food and nutraceutical applications [7], while polysaccharides exhibited a variety of biologically active compounds, including antioxidants [40] and stimulators of plant growth [41].

In the present study, we emphasized the role of temperature in determining changes in the amino acid and fatty acid profiles, under constant light at 100 µmol photons m^−2^ s^−1^, a light intensity typically observed in dense microalgal cultures at a depth of 3–4 cm [42]. Actually, temperature is recognized as a fundamental determinant of cellular function, directly influencing metabolism, enzyme activity, membrane fluidity, and protein structure. It governs the rate of biochemical reactions while maintaining the structural integrity of nucleic acids and proteins [43,44]. *Chlorella sorokiniana* cells grown at 40 °C under 100 µmoles photons m^−2^s^−1^ significantly altered the amino acid profile, increasing the relative abundance of arginine, lysine, tyrosine, and methionine and reducing levels of proline, leucine, and phenylalanine. Although *C. sorokiniana* is known for its thermotolerance, temperatures exceeding 40 °C can impair photosynthetic proteins, reduce productivity, and ultimately lead to cell death. High temperatures affect cellular metabolism by modulating enzymatic activity and the synthesis and degradation of specific amino acids. Similar responses have been reported in *Chlorella* spp. exposed to other stressors, such as copper, where increases in amino acids like glycine and alanine and decreases in others (e.g., isoleucine, leucine, valine, and arginine) were observed [45]. These findings indicate that amino acid profiles are highly dynamic and play an important role in stress acclimation by adjusting protein turnover and energy allocation. Total lipid content remained relatively stable across most treatments, with a maximum under low-light conditions at 25 °C, consistent with previous findings [46]. An increased content of α-linoleic acid was found in microalgae cultures grown at a sub-optimal temperature of 25 °C, reaching up to 14.6% of the total fatty acids in cells grown under 100 µmol photons m^−2^ s^−1^. Both changes in fatty acid and amino acid profiles underscore opportunities to gain further insights into the physiology and biotechnology of microalgae for their effective exploitation.

Despite decades of research, the average outdoor productivity of industrial microalgal strains, including *C. sorokiniana*, remains well below theoretical maxima. Identifying and mitigating factors limiting productivity is therefore essential. According to [47], achieving an annual biomass productivity of at least 25 g m^−2^ d^−1^—approximately double current average values—is necessary for economically viable biofuel production. However, such efficiencies are typically attained only under restricted conditions, namely, low light and optimal temperature, which are rarely sustained outdoors.

Technological solutions, such as vertical photobioreactors that dilute incident light, may help alleviate light stress [39]. Alternatively, thin-layer cascade systems, in which cells experience rapid light–dark cycles, have shown promise for maintaining high productivity [48]. In parallel with engineering approaches, the domestication of microalgae—analogous to strategies used in crop improvement—represents a realistic pathway to enhance strain performance. Selecting strains with enhanced stress tolerance and stable productivity could significantly improve annual yields in outdoor systems.

## 5. Conclusions

The results of this study highlight the importance of selecting robust *Chlorella sorokiniana* strains that can withstand combined environmental stresses, remarkably light and temperature, under outdoor cultivation conditions. *C. sorokiniana* displayed a broad temperature range for growth, spanning 20–40 °C under low light and 25–40 °C under higher light intensities. Growth was strongly inhibited under the combination of high light (200 µmol m^−2^ s^−1^) and suboptimal temperature (20 °C).

These findings suggest that preventing culture temperatures from falling below 20 °C is critical, especially in desert regions and temperate climates where such conditions are common. In thin-layer cascade systems, which involve relatively small volumes of culture per unit of surface area, one effective strategy is to store cultures in tanks overnight and resume circulation only when ambient temperatures are sufficiently high, thereby avoiding productivity losses [48]. In open-pond systems in colder regions, reducing nighttime mixing intensity can help limit heat loss. Mixing in outdoor ponds accelerates heat dissipation by disrupting surface thermal layers and enhancing forced convection, evaporation, and heat exchange with the surrounding air. While reducing nighttime heat loss may help maintain favorable temperatures, it can also increase respiratory rates, potentially reducing net biomass yield [49]. Therefore, decisions regarding nighttime temperature management should be tailored to local climatic conditions and system design. Overall, this study underscores the need for integrated strategies combining strain selection, physiological understanding, and cultivation system design to improve the reliability and productivity of *Chlorella sorokiniana* in outdoor environments.

## Figures and Tables

**Table 1:** Variations in protein, carbohydrate, and lipid of *C. sorokiniana* UTEX grown at six different temperatures and two light intensities (means ± standard deviation of 3 replicates). At both 20 °C and 45 °C under 200 μmol m^−2^ s^−1^, no growth occurred. Biochemical biomass composition was performed at the end of the experiment (8 days).

Temp. °C	20		25		30		35		40	
PFD μmol photons m^−2^ s^−1^	100	200	100	200	100	200	100	200	100	200
Protein (Lowry) (% DW)	35.60 ± 1.70 n.s.	n.d.	53.10 ± 1.89 ***	30.74 ± 1.02 ***	51.20 ± 3.80 ***	30.00 ± 3.60 ***	51.80 ± 2.50 ***	44.10 ± 1.60 ***	58.70 ± 1.90 n.s.	57.30 ± 1.50 n.s.
Carbohydrate (% DW)	39.42 ± 1.8 ***	n.d.	20.50 ± 1.84 ***	48.00 ± 2.20 ***	24.20 ± 2.13 ***	44.70 ± 2.10 ***	21.20 ± 2.13 ***	32.70 ± 1.06 ***	18.01 ± 1.80 n.s.	18.00 ± 2.30 n.s.
Lipid (% DW)	19.87 ± 1.10	n.d.	21.5 ± 1.17 ***	17.20 ± 1.20 ***	20.60 ± 1.62 *	20.90 ± 1.50 *	22.90 ± 1.74 ***	18.60 ± 1.10 ***	19.10 ± 1.10 *	19.70 ± 2.30 *
Total	95	n.d.	95	95.9	96.0	95.60	95.9	95.47	94.87	95.06

For each factor, asterisks indicate a significant difference between means, factorial ANOVA (Turkey’s test for statistical significance). n.s. = not significant (*p* > 0.05), * = (*p* < 0.05), *** = (*p* < 0.001). n.d. (not determined). The mean ash content was 4.6% of dry weight.

**Table 2:** Fatty acid profile of *C. sorokiniana* UTEX 1230 biomass grown at different temperatures and PFD = 100 μmol m^−2^-s^−1^. Biomass was harvested at the end of the 8-day growth experiment. SFAs, saturated fatty acids; USFAs, unsaturated fatty acids.

Growth Temp. (°C)	Fatty Acid Methyl Esters (FAMEs)				
	Palmitic C16:0	Oleic C18:1 n9	Linoleic C18:2 n6	α-Linoleic C18:3 n3	SFAs/USFAs
25	24.57 ± 0.49	6.38 ± 0.19	54.40 ± 1.63	14.63 ± 0.44	0.325 ± 0.028
35	29.51 ± 0.50	6.69 ± 0.20	55.52 ± 1.62	8.28 ± 0.23	0.418 ± 0.026
40	27.03 ± 0.49	7.75 ± 0.21	58.20 ± 1.64	7.00 ± 0.22	0.370 ± 0.026

**Table 3:** Amino acid profile of *C. sorokiniana* grown at different temperatures and exposed to an incident light irradiance of 100 µmol m^−2^ s^−1^. Data means 3 replicates.

Amino Acid	Growth Temperature			
	20 °C	25 °C	30 °C	40 °C
His	2.03 ± 0.04	2.22 ± 0.04	2.16 ± 0.08	2.09 ± 0.09
Ser	3.01 ± 0.09	2.92 ± 0.06	2.97 ± 0.06	3.01 ± 0.14
Arg	5.48 ± 0.16	5.09 ± 0.20	5.21 ± 0.15	6.29 ± 0.19
Gly	6.30 ± 0.15	6.97 ± 0.14	6.30 ± 0.14	6.24 ± 0.25
Asx	10.45 ± 0.31	10.11 ± 0.49	10.73 ± 0.39	10.36 ± 0.11
Glx	15.55 ± 0.46	14.89 ± 0.46	15.57 ± 0.35	15.30 ± 0.45
Thr	4.23 ± 0.08	4.07 ± 0.14	4.20 ± 0.03	4.35 ± 0.05
Ala	8.73 ± 0.08	8.75 ± 0.07	8.78 ± 0.06	8.58 ± 0.09
Pro	5.88 ± 0.16	7.03 ± 0.17	6.11 ± 0.15	5.69 ± 0.15
Cys	0.00 ± 0.00	0.00 ± 0.00	0.00 ± 0.00	0.06 ± 0.01
Lys	7.61 ± 0.22	7.82 ± 0.23	8.08 ± 0.27	9.53 ± 0.31
Tyr	0.87 ± 0.02	0.78 ± 0.01	0.77 ± 0.02	1.21 ± 0.03
Met	0.46 ± 0.01	0.39 ± 0.01	0.44 ± 0.01	0.76 ± 0.02
Val	6.98 ± 0.21	6.89 ± 0.20	6.80 ± 0.21	6.80 ± 0.22
Ile	4.96 ± 0.15	4.93 ± 0.20	4.86 ± 0.18	4.62 ± 0.15
Leu	10.90 ± 0.33	10.68 ± 0.31	10.69 ± 0.32	9.59 ± 0.29
Phe	6.58 ± 0.21	6.46 ± 0.20	6.37 ± 0.21	5.54 ± 0.19
Total	100.00	100.00	100.00	100.00

**His**, Hystidine; **Ser**, Serine; **Arg**, Arginine; **Gly**, Glycine; **Asx**, Asparagine or aspartic acid; **Glx**, Glutamine or glutamic acid; **Thr**, Treonine; **Ala**, Alanine; **Pro**, Proline; **Cys,** Cysteine; **Lys**, Lysine; **Tyr**, Tyrosine; **Met**, Methionine; **Val,** Valine; **Ile**, Isoleucine; **Leu,** Leucine; **Phe**, Phenylalanine; Referenced from international biochemical nomenclature guidelines.

## Data Availability

The data that support the findings of this study are available from the corresponding author upon reasonable request.
